# Development and piloting of a treatment foster care program for older youth with psychiatric problems

**DOI:** 10.1186/s13034-015-0057-4

**Published:** 2015-06-26

**Authors:** J. Curtis McMillen, Sarah Carter Narendorf, Debra Robinson, Judy Havlicek, Nicole Fedoravicius, Julie Bertram, David McNelly

**Affiliations:** School of Social Service Administration, University of Chicago, 969 E. 60th, Chicago, IL 60636 USA; Graduate College of Social Work, University of Houston, 110HA Social Work Building, Houston, TX 77204 USA; Washington University School of Medicine, Campus Box 1007, St. Louis, MO 63105 USA; School of Social Work, University of Illinois, 1010 W. Nevada Street, Urbana, IL 61801 USA; Research Consultant, Charlottesville, VA USA; St. Louis University School of Nursing, 3525 Caroline St, St. Louis, MO 63104 USA; Jackson County (Ohio) Board of Developmental Disabilities, 822 Sellars Drive, P.O. Box 607, Jackson, OH 45640 USA

**Keywords:** Foster care, Treatment foster care, Emotion regulation, Emerging adulthood

## Abstract

**Background:**

Older youth in out-of-home care often live in restrictive settings and face psychiatric issues without sufficient family support. This paper reports on the development and piloting of a manualized treatment foster care program designed to step down older youth with high psychiatric needs from residential programs to treatment foster care homes.

**Methods:**

A team of researchers and agency partners set out to develop a treatment foster care model for older youth based on Multi-dimensional Treatment Foster Care (MTFC). After matching youth by mental health condition and determining for whom randomization would be allowed, 14 youth were randomized to treatment as usual or a treatment foster home intervention. Stakeholders were interviewed qualitatively at multiple time points. Quantitative measures assessed mental health symptoms, days in locked facilities, employment and educational outcomes.

**Results:**

Development efforts led to substantial variations from the MTFC model and a new model, Treatment Foster Care for Older Youth was piloted. Feasibility monitoring suggested that it was difficult, but possible to recruit and randomize youth from and out of residential homes and that foster parents could be recruited to serve them. Qualitative data pointed to some qualified clinical successes. Stakeholders viewed two team roles – that of psychiatric nurse and skills coaches – very highly. However, results also suggested that foster parents and some staff did not tolerate the intervention well and struggled to address the emotion dysregulation issues of the young people they served. Quantitative data demonstrated that the intervention was not keeping youth out of locked facilities.

**Conclusions:**

The intervention needed further refinement prior to a broader trial. Intervention development work continued until components were developed to help address emotion regulation problems among fostered youth. Psychiatric nurses and skills coaches who work with youth in community settings hold promise as important supports for older youth with psychiatric needs.

## Background

This paper describes the development and piloting of a treatment foster care intervention program for older youth from the child welfare system with mental health challenges. Treatment foster care may be positioned to play a role in improving the outcomes of transition-age youth, potentially within both child welfare and mental health systems of care. Both systems have recognized service gaps in programming for transition-age youth with mental health challenges [1–3]. These service gaps may impede progress on the challenges and tasks of emerging adulthood in a first-world economy, such as graduating high school, starting college, gaining employment experience and avoiding incarceration. Research on early adult outcomes from young people served in foster care, mental health and special education systems have demonstrated poor functional outcomes in early adulthood, especially in the areas of employment and incarceration [1, 3–6]. For youth in foster care systems, educational attainment is also low [1].

In addition to leaving foster care with mental health problems [7, 8], older youth in the foster care system typically have experienced a high number of living situations, including congregate care settings [9]. No placement type, including residential care or treatment foster care, seems to be able to stop the cycle of failed placements and replacements [9]. Many youth who remain in the foster care system as older teens entered the foster care system earlier in their teenage years and compared to children who entered earlier, they are more prone to enter a residential program (as opposed to living in a foster home) and less likely to achieve permanency through adoption or guardianship with relatives [1, 8, 10]. While they typically receive mental health services while in the foster care system [11], mental health service use drops precipitously once they leave the foster care system [12].

While most experts believe that older youth (ages 16–21) are best served in family settings, residential group treatment in child welfare systems is common [13]. About 15 % of youth served in the foster care system in the U.S. are served in group home or institutional settings [14] and for older youth, this number is higher, sometimes as high as 60 % [9, 11]. The research base for residential group treatment effectiveness is not robust [13]. Yet, there is typically correspondence between the level of a young person’s functioning and where he or she resides in the care continuum [7, 15, 16]. Stepping youth down to community based settings without the benefit of improvement in functioning is difficult [17], meaning young people in the foster care system often experience a sudden and harsh transition from institutional living to living on their own [18].

A primary reason to serve older youth with mental health problems in family homes rather than residential group treatment settings is that it is difficult to align residential group settings with the conditions that are thought to promote positive development. Walker and Gowen [19] summarized the key features of settings that are thought to promote development of young people in their transition to adulthood:

“Such environments are psychologically and physically safe; they provide connection to prosocial adults and peers; they allow for opportunities to build skills; and they provide a balance between structure and flexibility, so that while there are clear expectations, there are opportunities for young people to set goals and make decisions and plans about how to react to those goals.” (p. 8)

In contrast, Scannapieco, Connick-Carrick and Painter [20] found that services for older youth in foster care were characterized as lacking in respect for individual youth, lacking youth involvement in decision-making, lacking real-life practice for skill development and lacking opportunities to forge permanent connections.

### Treatment foster care as a step-down option

Treatment foster care programs (also called specialized and therapeutic foster care) serve as alternatives to residential group treatment. While treatment foster care is commonly used as a placement option for child welfare and child mental health systems, only two models have been empirically tested in randomized trials. In a single trial, Farmer et al. [21] showed that training treatment foster parents and treatment foster care supervisors in well-specified behavior management strategies could result in short term improvements for youth placed through the child mental health system in treatment foster homes. In this study, fostered youth were 13-years old on average and had been living in their current treatment foster homes for 20 months before randomization by agency to receive Farmer’s behavioral training or treatment as usual (TAU). Favorable treatment effects were seen for three outcome measures at six-month follow-up (for total difficulties, number of types of problems and strengths), but these faded to non-significance at the 12-month follow-up for two of the three measures (total difficulties and strengths).

The behavioral training in this program is largely adapted from Chamberlain’s Multidimensional Treatment Foster Care (MTFC) intervention, which is based on the same behavioral principles as Farmer’s intervention. MTFC involves a large team, an extensive behavior management system, family treatment to support reunification and high levels of foster parent support [22]. MTFC has become popular in the juvenile justice system as an alternative to youth incarceration, but has not been widely adopted in child welfare or mental health systems.

Treatment foster care may be one platform to step down older youth with mental health challenges from residential settings to community living, but the suitability of the two evidence-based programs for this population is unclear. MTFC was chosen as the basis for an intervention for older youth with mental health challenges over Farmer’s program for two reasons. (1) It is more far more intensive that Farmer’s program. Older youth with mental health challenges living in residential centers were thought to require an intense intervention if they were to step down to family living. (2) It has much more evidence supporting its effectiveness [23]. In randomized trials with adolescents primarily served in the juvenile justice system, MTFC has outperformed comparison conditions across a wide variety of outcomes including behavior problems, criminal offenses, returns to family, incarcerations and early pregnancy [24–29]. Therefore, a developmental project was designed to use stakeholders to examine MTFC’s suitability for this population, make needed alterations, and pilot a resulting treatment foster care model.

### Program development

A local U.S.-based private child welfare foster care agency was recruited to participate with academic partners in intervention development. The agency was recommended for the pilot by the director of the regional public child welfare authority for three reasons: it had a history of innovation, operated on a capitated payment structure in line with the goal of stepping youth down from more expensive to less expensive treatment, and had a population of youth designated to be of high need by the child welfare authority on the basis of their placement histories. Five members of the intervention development team were trained in MTFC from TFC Consultants, Inc. Then, a panel of local stakeholders was convened to consult with national experts and determine whether MTFC needed adaptation to meet the needs of older youth in the child welfare foster care system with mental health challenges who were currently being served in a higher level of care. The stakeholder panel consisted of the group recently trained in MTFC, two foster parents, two foster youth, a doctoral student in social work with a long history of child mental health experience and a national expert in services for older youth in foster care. Additional consultants were hired and used as needed, including experts in residential group treatment and cultures that promote youth development, psychoeducation for mental disorder and psychiatric nursing.

The stakeholder team determined that MTFC would not meet the needs of older youth in the child welfare foster care system with mental health challenges as designed and traditionally implemented. The MTFC features that led to this conclusion included four foci that MTFC *lacked* and were considered important for the population by the stakeholder team. These included:

1. the lack of specified psychiatric components, including the facilitation of psychiatric care continuities and transitions, ways to interact with psychiatric providers, psychoeducation for mental health problems and preparation for youth to take a more active role in their mental health care;
2. a lack of focus on acquiring and practicing life skills in areas such as employment, transportation, shopping, *etc.*;
3. a lack of focus on future planning for education, employment and housing; and
4. a general lack of youth voice in treatment.

Further justifications for moving away from MTFC as the model program for older youth were found in the MTFC focus of family work on return home; a strict behavior management system maintained throughout the youth’s time in the program; and an emphasis on documenting the whereabouts of MTFC youth at all times.

After consulting with the MTFC developer, the team decided with her permission to use the basic structure and many strengths of the MTFC program and write new intervention manuals, with the understanding that the new intervention would not be called MTFC or referred to as a variant of MTFC. Intervention manuals were written by the project investigator, one program supervisor and a doctoral student. The other stakeholders had two opportunities to review and improve the manuals as they were developed. The resulting manuals comprised Treatment Foster Care for Older Youth (TFC-OY). TFC-OY borrowed the multiple team-member approach of MTFC, but with team member roles adapted and others created. These roles and their relationship to MTFC are shown in Table 1.

**Table 1:** Roles in the Treatment Foster Care for Older Youth (TFC-OY) interim intervention

TFC-OY role	Envisioned purpose	Relation to MTFC
Program supervisor	To coordinate, supervise and individualize the young person’s treatment program and to serve as the communication hub among the team members.	Similar to MTFC.
Treatment foster parent	To encourage, support and supervise the young person.	Similar to MTFC.
Life coach	To support the young person’s adjustment in the program by (a) helping the young person build social skills, (b) plan-fully prevent problems, and (c) to help prepare for the future by acting as the young person’s chief partner in planning and understanding their mental health issues. For youth unable to plan for the future due to unresolved trauma, the Life Coach could focus on helping the youth prepare for trauma treatment.	The MTFC therapist focused on only some of these activities (a and b).
Psychiatric nurse	To help clarify young people’s existing mental health issues and treatment options.	Newly developed role.
Family consultant	To focus on building connections with the young person’s family members or other adults that will love, support and respect them.	Different focus than MTFC’s family therapist role, which focused on reunification.
Skills coach	To support young people’s adjustment and success by orientating them towards socially acceptable activities within the community and helping them learn and practice life skills *in vivo* in the community.	MTFC’s skills coach role does not include life skills preparation.

Among the most substantial changes were the following.

1. A role for a psychiatric nurse was created to assist in clarifying mental health diagnostic status and medications and to facilitate continuity of mental health care as youth transitioned into treatment foster care and across foster care homes. This role was configured as a part-time role, no more than 10 h per week per team. In the ensuing project, a master’s level psychiatric advanced practice nurse was used.
2. A family consultant role was designed to build community supports for youth to live more independently. The two main activities were family finding [30] methods to reconnect youth with people from their pasts who could be resources for them and use of the permanency pact [31], a tool to build specific supports for youth from a specified menu.
3. The role of a master’s level life coach was created (in lieu of a therapist) to assist youth in the transition to the foster home and in preparation for their next steps in the community. The role was initially intended to start dialogue about youth interests and hopes and move toward planning for the future and then provide psychoeducation about the young person’s specific mental health issues following a set protocol. The life coach met weekly with young people and billed Medicaid for this service. The two life coaches who worked on the project were experienced master’s level therapists.
4. A new point and privilege system was developed for use in the foster home, with three phases designed to wean youth off of daily behavioral management charting. In the first phase, daily privileges were earned from the prior day’s point total, with the young person’s behavior rated by foster parents in ten areas (each worth ten points). Behavior, points and privileges were reviewed with the young person each evening. In the second phase, the points were eliminated, with privileges for the next day determined after an evening review of the ten domains (with no points assigned). In the third phase, a more general daily review between youth and foster parent was encouraged, but privileges were not determined on a daily basis.
5. Skills coaches (different from life coaches) who worked with youth outside the foster home at least weekly, focused on independent living skill acquisition and healthy activities in the community. Youth identified areas in which they wanted to participate in the community in work with their life coach and the skills coach worked with the team and youth to develop those opportunities in community settings. In addition, the skills coach provided one-on-one coaching in independent living skills such as shopping, budgeting, job search, job interview preparation and transportation. Skills coaches in the ensuing project possessed bachelor degrees and were students in a master of social work program.
6. A 16-h TFC-OY foster parent training was created and manualized that emphasized description of the young people foster parents would be asked to work with, an overview of the program, noticing problem and cooperative behaviors, encouraging youth, the point system, teaching independent living skills, and creating opportunities for youth.

Several features from the MTFC model were retained with modest adaptation. 1) The program supervisor ran the weekly team and foster parent meetings and was responsible for communication within the team and with the young person’s family support team and agency case manager. This person was available *via* phone to foster parents on nights and weekends. 2) Foster parents met weekly with each other and the program supervisor to identify problem behaviors to target and develop strategies to be used in the home to address these concerns.

Each role was specified in detailed manuals. Since foster care is a 24/7 service, it is not possible to provide protocols for every contingency that can arise. Staff were therefore to be guided not just by the manuals, but by guiding philosophies. These were originally developed by the project investigator in consultation with the project coordinator and then vetted and amended by the intervention development team. They were: to serve youth in families and communities, provide positive developmental opportunities, foster connections, encourage and enrich vital skills, limit access to negative peers, involve young people, have fun, individualize services, communicate among parties, recognize young people when they do well, plan-fully prevent problems, and help young people understand their mental health issues.

Consistent with policy created by the state child welfare authority, youth retained their private agency case manager and their family support team. The family support team in this context was a group of adults (and the youth) who were consulted on case decisions at least once monthly including on placement decisions and treatment directions. It is designed to promote better decision making, family involvement, and continuity of care.

### Pilot study research questions

Once an interim version of the intervention was developed, an intervention pilot was conducted concurrent with a small mixed methods study. The study was designed to address a number of questions. Feasibility questions focused on recruitment of youth and foster parents, randomization, and tolerance of the intervention and research protocols. Programmatic questions were also addressed. What would stakeholders think of new intervention components and roles? Were programmatic changes needed before moving forward with a larger trial?

Assessing how participants respond to interventions clinically is a known thorny issue for pilot research. Pilot researchers have been admonished “to bravely accept the limitations of a pilot study” (p. 628) [32], to focus on feasibility, and not to use them to gauge efficacy or calculate effect sizes for a larger trial [32, 33]. Understanding these same limitations, Fraser, Richman, Galinsky and Day [34] suggested using pilot studies to refine interventions, to “describe the process of interaction between the practitioners and participants” (p. 82), with a focus on understanding participants’ reactions to interventions. Thomas [35] suggested that pilots could identify cases with satisfactory and unsatisfactory outcomes and while not testing outcomes, they could examine what some of the outcomes appear to be. With these warnings in mind, we avoid testing difference between groups, while still examining outcomes related to maintaining youth in community settings (out of locked settings), changes in youths’ mental health symptoms over the course of the study, and progress on functional indicators such as employment and school completion. In addition, qualitatively, we explore whether stakeholders think there were clinical successes.

## Methods

The mixed-methods pilot used a randomized design with a focus on qualitative inquiry. Table 2 matches the research questions described above with the methods used to assess them. With sample size not determined by the need for inferential statistical testing, it was determined by pragmatics [33]. A small pilot was chosen. One treatment team delivered TFC-OY over 18 months and a research project was wrapped around it. While 18 months is long for a pilot effort, treatment foster care is an unusual and often lengthy intervention. We wanted to see how the program played out over a substantial period of time. Approval to conduct the research was obtained from the state child welfare authority and a university IRB.

**Table 2:** Research methods by research question

Research Question	Methods to address the question
Would randomization to less restrictive care be allowed?	Tracked care manager and family support team decisions in database.
Could foster parents be recruited to serve youth stepping down from residential treatment?	Kept track of foster parents who completed training in the TFC-OY model and who had youth placed in their home.
How would foster parents and staff tolerate the intervention?	Qualitative interviews with foster parents 2 months into placement and at service termination. Qualitative interviews with staff at end of program.
What would stakeholders think of the innovations in the treatment model?	Qualitative interviews with stakeholders at end of intervention.
How would youth respond to the intervention clinically?	Structured interviews with TFC-OY youth at baseline and 6, 12 and 18 months later tracked mental health symptoms, hospitalizations, incarcerations, employment and educational milestones. Qualitative interviews with youth, staff and care managers asked about clinical successes and failures.
Were program changes needed?	Qualitative interviews with stakeholders at end of intervention.

### Participants

Youth were eligible if they (1) were 16 to 18 years old, (2) were in state child welfare custody and served by the private agency, (3) had been hospitalized for psychiatric illness in the past year or were receiving psychotropic medications; (4) were residing in a residential facility, (5) had been in the foster care system for at least 9 months and (6) had a full scale IQ of 70 or greater. Administrative databases identified 96 potentially eligible young people based on age and placement data. Care managers were approached by the project director to determine if youth met additional study criteria and 46 of the 96 did. If the youth was eligible and the care manager provided informed consent to randomization and the other research protocols, youth were approached for informed assent and an initial in-person structured research interview was conducted. Foster parents, program staff and case managers were consented prior to their research interviews.

After the baseline interview, youth were matched into pairs based on their interview-derived or official agency mental health diagnoses. If family support teams approved both pairs of matched youth for randomization, youth from the pairs were randomized to TAU or TFC-OY conditions. Randomization was conducted by a statistician external to the study. Three random numbers were generated, one for each youth in the pair and a third number for assignment. The youth with the random number closest in absolute value to the third random number was assigned to the TFC-OY condition. The TFC-OY youth were removed from their residential treatment center and placed to a treatment foster care home as soon as possible. TAU youth remained in their residential treatment placement.

### Measurement

The initial (pre-randomization) interview included portions of the Diagnostic Interview Schedule Version IV [36] that assessed criteria for lifetime and past year psychiatric diagnoses and a measure of mental health symptoms, the Brief Symptom Inventory (BSI) [37]. The interview assessed for maltreatment history using the Child Trauma Questionnaire [38] and items on sexual abuse adapted from Russell [39]. Reading levels were assessed with the Woodcock Johnson III Passage Comprehension protocol [40]. Youth randomized to TAU or TFC-OY received additional structured interviews at 6, 12 and 18 months. These interviews assessed placements and placement changes, mental health symptoms using the BSI, education milestones, and employment experiences. Youth reported the number of days in the past 180 that they were in locked facilities, attending school, and worked in paid employment. All youth completed all interviews. These interviews were conducted by a master’s level researcher who was not blind to study condition.

### Qualitative interviews

Qualitative interviews of youth in the TFC-OY condition were conducted two months after initial placement and at the end of the program. Interviews were conducted by a postdoctoral fellow and a doctoral student, both trained in qualitative interviews. Qualitative interviews with youth focused on experiences with and opinions of TFC-OY program components. Sample questions and prompts included the following. “Tell me about your experience with this part of the program.” “What do you like about it?” “What do you not like about it?” “What could be done differently to make this part of the program better?”

Qualitative interviews with foster parents were conducted two months after placement and at the end of the placement or the end of the program. Foster parents were asked about successes, how the provided training helped or did not help them foster the youth in their home, what things the staff did that were found to be helpful and what could be done differently to make the program better? Qualitative interviews with TFC-OY staff members and youth’s foster care case managers were conducted at the end of the program. Questions focused on challenges, successes and ways to improve the program. All qualitative interviews were audio recorded and professionally transcribed. Qualitative interviews lasted from 20 to 90 min.

### Analyses

Quantitative analyses were descriptive and sometimes involved looking at individual results over time. Content analysis [41], based on straightforward analytic questions, was the qualitative analytic approach. This approach examines language content and intensity in a subjective interpretation of classifications, themes and patterns. The focus was mainly on classification (*e.g.*, what did the stakeholders like?). Five members of the university-based research team analyzed the qualitative data in consultation with a qualitative methods consultant.

## Results

### Would stakeholders allow randomization?

**Fig. 1:**
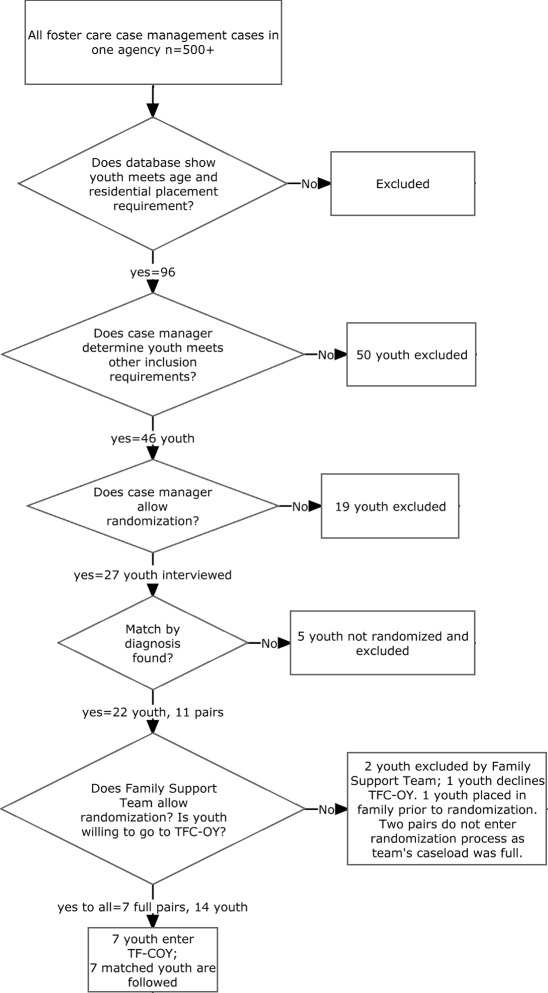
Sampling, consent, randomization and matching

The child welfare authority would allow randomization to a Treatment as Usual Condition (TAU) or TFC-OY if the youth’s care manager would consent to it, the youth would assent to it, and the youth’s family support team would support it. But, the degree to which the parties would find randomization into a treatment foster home acceptable was not known. Figure 1 shows the outcome of sampling, consent and randomization procedures. Of the 46 eligible youth, care managers chose to disallow randomization for 19. Reasons were (a) that plans were already in place to move youth from a residential center placement to a family or community placement (*n* = 8); (b) youth behavior was seen as too severe for a family placement (*n* = 7); (c) youth parents were placed with their children in the residential program (*n* = 2); (d) youth was court ordered to residential center placement (*n* = 1); and (e) care managers reported that youth would not agree to live with a family (*n* = 1).

For those whose care managers approved randomization and who were matched to another youth by diagnoses, family support teams were convened to decide whether randomization would go forward. One team thought the youth’s emotional and behavioral problems were too acute. Another team thought that the youth should be placed in a juvenile justice program. In two cases, the youth decided against randomization at this point. One youth expressed a desire to remain at the residential program because the youth liked it there. Another youth thought that he could reach his desired placement – a transitional living program – quicker if he remained in the residential program. One youth was placed in a foster home outside of the pilot program prior to randomization.

Eight pairs of youth were randomized to the TFC-OY or TAU conditions. Seven of the eight TFC-OY youth were placed in foster homes, while one youth decided not to be placed after meeting potential foster parents. Of the seven study pairs matched by diagnosis, three were matched based on bipolar disorder, two on basis of depression disorders, and two on disruptive behavior disorders. Thirteen youth completed the first diagnostic interview, but were not part of the intervention or TAU group. They either were not matched by diagnosis or one or both pair members declined further participation or they were not needed to complete a full TFC-OY caseload. We were unsure what the ideal caseload size would be and as part of the pilot, we allowed the program staff to tell us when they thought they had reached capacity. This happened when a caseload size of seven was reached. This coincided with a time when some young people placed earlier in TFC-OY homes began experiencing more behavior problems. It is unclear whether the team could have handled a slightly larger caseload size if youth were added more gradually to the caseload.

### Further description of the sample

Table 3 shows descriptive statistics for the seven youth in the TFC-OY condition, the seven youth in the TAU condition and the 13 youth who completed the screening interview and were not subsequently followed by the research team. We included baseline information on youth not in the small randomized conditions to allow readers to compare youth to a slightly larger group of youth with the same eligibility requirements. Participants were veterans of the foster care system with many prior placements, IQ scores typically a standard deviation below the mean, and reading grade equivalency scores several grades below grade level.

**Table 3:** Description of the sample

	TFC-OY	TAU	non-assigned
	(*n* = 7)	(*n* = 7)	(*n* = 13)
Female gender	5 (71 %)	5 (71 %)	7 (54 %)
Physical abuse history	4 (57 %)	4 (57 %)	4 (29 %)
Physical neglect history	2 (29 %)	1 (14 %)	3 (21 %)
Sexual abuse history	6 (86 %)	2 (29 %)	5 (38 %)
History of psychiatric hospitalization	6 (86 %)	7 (100 %)	9 (69 %)
Psychotropic medication at first interview	7 (100 %)	7 (100 %)	9 (69 %)
	Mean (SD)	Mean (SD)	Mean (SD)
Age at first interview	17.19 (.63)	17.25 (.93)	16.83 (.70)
Prior number of placements	13.85 (8.86)	10.57 (9.41)	7.92 (3.66)
Full Scale IQ in case record	83.86 (6.28)	81.29 (14.67)	79.5 (7.78)
Woodcock Johnson Passage Comprehension Recognition			
Grade Eq.	4.84 (1.93)	5.96 (5.99)	7.11 (3.13)

### Could foster parents be recruited to serve youth stepping down from residential treatment?

One feasibility concern was that youth would be randomized to the TFC-OY condition, but that foster parents would not be found to work with them. This was not a problem. All youth randomized to TFC-OY were placed in TFC-OY trained foster homes. Seven TFC-OY youth were placed into a total of 10 different homes (including re-placements), with 13 trained foster parents (three two-parent families and seven single parent families). Foster parents ranged from new to fostering to very experienced.

### How would foster parents and staff tolerate the intervention?

A second feasibility worry was that the TFC-OY intervention would be difficult for foster parents to tolerate. This was confirmed. In addition, some staff found the work stressful. In weekly meetings and in the qualitative research interviews, foster parents reported that the youth were extremely difficult to parent. Despite training that focused on the needs of youth with psychiatric problems, the foster parents reported being surprised by the amount of emotional volatility in the young people they served, the low levels of what they perceived as emotional maturity, and high needs for monitoring and supervision. The following quote from a foster parent is exemplary. “It is challenging every day because I just have to pay attention to her moods more. The hardest thing is that I have to monitor her so closely and I have to watch what I say.” No parent or youth described an extended period of time when life settled into a comfortable routine. It always felt like stressful work to the foster parents.

The experience was not easy for the TFC-OY staff either. One Life Coach was surprised by the low level of emotional functioning of youth in an office setting.

“It seems like all at once, the kids started being very chaotic and disrupting things all over the place, and everyone was coming into my office, all in a row. Boom, boom, boom. And it was just chaos, chaos, chaos, chaos. Crisis. Running away from appointments. Breaking things. And it was for a month straight.”

### What would stakeholders think of the innovations in the treatment foster care model?

The skills coach component was uniformly appreciated by foster parents, the program supervisor and the youth. When asked about the skills coach component, the youth tended to report things the coach had done for and with them that were related to positive youth development.

“She took me outside and she helped me find a job. She took me out to eat. She helped me get my driver’s license. She helped me get my permit. Helped me with my homework. She helped me learn how to make a grocery list, pay bills, audit. She helped me with a lot of things.”

Multiple stakeholders commented on the positive relationships that youth developed with their skills coaches, as exemplified in this quote from a staff member.

“They’ve been able to build a relationship with the kids that doesn’t have any strings attached. The kids look at them as somebody who’s on their side and doesn’t want anything from them.”

A second component that drew positive comments from stakeholders was that of the psychiatric nurse [42]. Care managers appreciated the medication and diagnostic review provided by the nurse. They provided numerous examples of how they used this review and knowledge in their interactions with mental health providers. While some youth did not understand why they were receiving psychoeducation about their mental health problems from a nurse, others greatly appreciated it, explaining that it changed how they monitored their symptoms and how they approached their psychiatric providers.

The role of the life coach was a difficult one to execute. Initially, the role was focused on interpersonal skills the youth needed to succeed in the foster home, but was later supposed to involve life planning and psychoeducation. Two life coaches worked in the program and both found their role frustrating.

“To talk with them about school and work and STDs and their grief issues and their placement issues and what they did in school and their upcoming court hearing….you can’t do all that so it was…at times it was a little overwhelming to try to basically do what I thought I was being asked to do.”

The family consultant role was less well received. The family consultant made many unsuccessful efforts to re-engage biological relatives and other nominated individuals into the lives of youth in TFC-OY and executed one successful effort, involving an older sibling. The role was also expensive (using a master’s level mental health professional). In the end, the principal investigator concluded that the family consultant role would be eliminated going forward and that needed family work would be conducted by the program supervisor.

### How would youth respond to the intervention clinically?

In this section, descriptive information on outcomes is provided for the youth served in the TFC-OY program. Also provided are numerical descriptors of the youth in the TAU condition, although the sample sizes are not large enough to allow statistically valid comparisons.

#### Would TFC-OY youth be maintained in community settings?

The conclusion was no. The program was unable to maintain youth in community settings throughout the pilot. Over the 18 study months, five of the seven youth spent time in a locked facility, ranging from 14 to 106 days. On average, the seven TFC-OY youth spent 45.85 nights (SD = 42.91) in a locked facility (8.38 % of all nights). Over the 18 study months, the seven TAU youth spent an average of 12.57 nights (SD = 22.94) in a locked facility (2.3 % of the time). Two of the seven spent time in a locked facility, with a range from 30 to 58 days.

#### Would the trajectory of youth mental health symptoms change in response to the intervention?

TFC-OY was not designed to decrease mental health symptoms. It was designed to see if youth with high levels of mental health symptoms could be served in community settings without substantial mental health deterioration. Among the seven TFC-OY youth, Global Severity Scores on the BSI increased for one youth, decreased for one youth and remained relatively flat for five youth. Among youth in TAU, one had dramatically increasing scores. The other six scores remained flat.

#### Would youth show progress on functional indicators such as employment and school completion?

Only two of the seven youth assigned to the TFC-OY condition had any paid employment prior to study inception. Three of these seven youth earned income during the course of the study, with only one youth earning money in each 6 month reporting period. Six of the seven youth in the TAU condition had prior employment experience. Six of the seven TAU youth earned money from employment during the course of the study, with none earning money in each reporting period. The most money earned by any youth over the 18 months of study was $4640.

At the first interview, none of the young people had graduated from high school or completed an equivalency diploma. Of the seven youth in TFC-OY, at 18 months, two graduated high school, one was attending a community high school, one was in a equivalency diploma program, two were in treatment-oriented schools and one was not attending school and had not graduated. Of the seven youth in the TAU condition, at 18 months, four had graduated from high school, one was attending a public high school, one was in an equivalency diploma program, and one was attending a treatment oriented school.

### Qualitatively, did stakeholders think there were clinical successes?

Stakeholders perceived qualified clinical successes. One example quote is from a caseworker who thought that the youth’s participation was beneficial even though her stay in an initial foster home placement lasted only a few months.

“I think what was most helpful for her out of the experience was just knowing that she could be in a home, and that she realized that she had more control over her behavior than she thought she did. She’d say, ‘You know, I’m crazy, I can’t live in a foster home.’ That kind of stuff. And so I think her being in that foster home, even though it was four months, she was like no other time I’ve seen her.”

Another qualified success was described by this foster parent, who saw substantial improvements in functioning in a youth she served. “She improved so much in her attitude toward others. It doesn’t mean that she was without problems at the end, but it did mean that she seemed to start to get it. And that is the type of thing you feel really good about [43].”

### Were program changes needed?

Since it was decided that it was permissible to alter the intervention mid-pilot in order to have an intervention worthy of testing at the end of pilot period, two modifications to the protocols were made several months into the intervention: 1) redefined roles for team members; and 2) efforts to address emotional dysregulation.

Some of the life coach’s responsibilities were off-loaded to other team members. The skills coaches became responsible for helping youth plan for more independent living and the psychiatric nurse became responsible for providing psychoeducation about mental health problems. These modifications were considered successful, as viewed by stakeholders in qualitative interviews at the end of the project.

Most glaring was the need to develop intervention components to address youth emotion regulation problems. Six of the foster parents interviewed qualitatively reported that the young people served in their homes experienced severe emotional outbursts; typically youth were seen as quick to become emotional and remaining emotionally volatile for substantial periods of time. In their qualitative interviews, foster parents used words like “fuming mad,” “raging mad,” “explosive,” “just rage,” “outbursts,” “out of control,” and “blowing up.”

This was seen and reported by program staff as well. These are the words of one of the life coaches who phrased the problem as one related to borderline personality issues and the possibility of incorporating components from a treatment for borderline personality disorder, Dialectical Behavior Therapy or DBT, known for addressing emotion regulation problems [44].

If they have Axis Two with Cluster B stuff going on, I don’t think that the families are prepared for what kind of emotions that can bring up… So I don’t know if there needs to be some sort of training for the foster parents, training to know how to handle that. Have the foster parents go through some sort of DBT training themselves? So that they’re at least speaking the same language to remind them to use their skills.

During the last six months of the pilot, TFC-OY staff explored the potential of using processes and materials from DBT in TFC-OY to address youth emotion regulation problems. Staff received initial DBT training from a certified trainer and a DBT skills group was mounted with the foster youth to teach interpersonal effectiveness and mindfulness skills. The groups were well received by youth who attended them, but attendance was a problem, mostly due to logistics, such as distance from youth placements to the group site, work schedules, and transportation issues. By the end of the pilot, the intervention team concluded that any future trials or implementation of TFC-OY should be delayed until new intervention components were developed to address emotion regulation problems.

## Discussion

The mixed-method small pilot of a treatment foster care intervention for older youth with high levels of psychiatric need was informative on many levels. It addressed a number of feasibility issues and helped identify program components that worked and those that needed to be re-worked. The pilot was able to address many of the research feasibility aspects suggested in the literature, including the feasibility of measurement, recruitment, randomization, and retention [32, 45]. While more than sufficient to populate a pilot study, recruitment efforts were only modestly successful. Decision makers and youth themselves declined randomization in many cases. Future efforts to recruit youth from residential programs and randomize them to community settings may need a large pool from which to draw youth to populate larger studies. Pilot results suggested that foster parents could be recruited to serve these youth, and that youth were tolerant of the data collection protocols.

While pilot trials are not designed to assess whether interventions work, results can be dissected to look for signs that an intervention may have the potential to work. Here, results were markedly mixed. It was not our expectation that mental health would improve as youth left 24-h residential programs for residential treatment, but that mental health would not deteriorate as youth moved into the community. In this study, mental health symptoms mostly remained stable over time for most youth in both conditions. The fact that we had but one baseline measure before three follow-ups meant, however, that we could not fully capture symptom trajectories.

Most concerning, there was little evidence that the intervention was keeping youth out of locked facilities. In this underpowered pilot, youth in TFC-OY appeared to spend more days in locked settings. Too often, crises escalated to the point where a care manager would decide to use a short term psychiatric placement as a crisis management tool. The stable BSI scores over time for both groups, however, suggests that it is not that the TFC-OY program was leading to deteriorating mental health, but that the 24-h residential programs in which the TAU group remained may have been better equipped than foster homes to handle emotional outbursts without resorting to in-patient psychiatric admissions and better able to limit criminal involvement. Yet, clinical successes were described by stakeholders in qualified terms.

Not surprisingly given the small sample size, randomization to create similar groups likely failed, in that the TFC-OY group appeared to have more severe abuse histories, lower reading levels and a greater number of prior out-of-home placements than other youth in the study. This further complicates conclusions that can be drawn from the clinical and functional outcomes reported here. TFC-OY foster parents may have been asked to deal with a group of youth particularly prone to emotional dysregulation and hospitalizations.

The pilot also revealed aspects of the intervention program that were viewed as successful and could be of value in other service configurations for emerging adults with mental health challenges. The role of the psychiatric nurse was considered so successful that a follow-up team has manualized additional nurse functions to create a more comprehensive role for psychiatric nursing in foster care agencies. The role of the skills coach, originally a feature of MTFC and reconfigured here to deal with the development of independent living skills and life planning, was uniformly viewed as helpful by stakeholders. This was the least expensive program role (graduate students in social work were hired and paid $10 per hour). This role has the potential to be integrated inexpensively into other programs with emerging adults with high levels of psychiatric need as a means to provide opportunities for productive development in the community.

Most importantly, the qualitative portions of the study were successful in identifying programmatic concerns that needed to be addressed. Some were addressed in the context of the pilot, as roles were adjusted. However, as the pilot project ended, it was decided that the program was not yet worthy of dissemination or further testing in a larger trial because of the need for a new intervention component that addressed youth emotion regulation difficulties. After the pilot ended, the first author worked with an additional team and additional agency (including youth and caregivers) to develop intervention components for youth and their foster parents to address emotion regulation issues. This component program, Handling Intense Emotions (http://handlingintenseemotions.com), is a blended intervention, combining digitized material and in-person facilitation. It provides psychoeducation to youth and caregivers about intense emotion episodes, including information on how they typically start and end, and how some youth end up with emotion regulation problems. It provides youth skills to employ when distressed, including concrete actions and two types of cognitive reappraisal. It also provides training for foster parents on how to prompt youth to use these skills. It teaches foster parents helpful things to say to youth while they are in the midst of an emotional outburst, primarily focusing on how to pair statements that validate youth’s emotional states with other things that need to be said, such as setting limits. Finally, it employs a specific model of meditational problem solving, to help resolve situations that could otherwise lead to ongoing emotional distress. These components will require their own pilot testing before the TFC-OY model is further tested. With the intervention reconceived, the team’s energy and confidence will carry forward into the next phase. The reformulated version of TFC-OY includes a program supervisor, foster parents, skills coaches, a psychiatric nurse, a life coach and a curriculum on emotion regulation for youth and foster parents that will be completed prior to placement and reinforced by the team members trained in the same curriculum. The life coach role is reconceived as assisting young people in their application of emotion regulation skills learned in the blended learning curriculum.

## Conclusions

The results point to the need for rich protocol development efforts for populations with as many and varied needs as those of emerging adults with mental health challenges. The programming required several adjustments, mid- and post-pilot. This speaks to the need for iterative processes in intervention development and efforts to capture stakeholder reactions and input. Programs serving this population need to assure that young people are (a) provided opportunities in the community, (b) while providing competent psychiatric treatment and (c) addressing functional issues across a range of settings. Treatment models with skills coaches that work with youth in community settings, psychiatric nurses who help manage transitions and provide psychoeducation, and program modules that address emotion dysregulation may hold promise in meeting these needs.

## Abbreviations

BSI: Brief symptom inventory
DBT: Dialectical behavior therapy
MTFC: Multi-dimensional treatment foster care
TAU: Treatment as usual
TFC-OY: Treatment foster care for older youth
